# The distribution of climbing chalk on climbed boulders and its impact on rock‐dwelling fern and moss species

**DOI:** 10.1002/ece3.6773

**Published:** 2020-10-01

**Authors:** Daniel Hepenstrick, Ariel Bergamini, Rolf Holderegger

**Affiliations:** ^1^ Institute of Integrative Biology ETH Zürich Zürich Switzerland; ^2^ WSL Swiss Federal Research Institute Birmensdorf Switzerland; ^3^ Institute of Natural Resource Sciences ZHAW Zurich University of Applied Sciences Wädenswil Switzerland

**Keywords:** bouldering, bryophytes, cliff ecosystem, human disturbance, magnesia, magnesium carbonate, plant conservation

## Abstract

Rock climbing is popular, and the number of climbers rises worldwide. Numerous studies on the impact of climbing on rock‐dwelling plants have reported negative effects, which were mainly attributed to mechanical disturbances such as trampling and removal of soil and vegetation. However, climbers also use climbing chalk (magnesium carbonate hydroxide) whose potential chemical effects on rock‐dwelling species have not been assessed so far. Climbing chalk is expected to alter the pH and nutrient conditions on rocks, which may affect rock‐dwelling organisms. We elucidated two fundamental aspects of climbing chalk. (a) Its distribution along nonoverhanging climbing routes was measured on regularly spaced raster points on gneiss boulders used for bouldering (ropeless climbing at low height). These measurements revealed elevated climbing chalk levels even on 65% of sampling points without any visual traces of climbing chalk. (b) The impact of climbing chalk on rock‐dwelling plants was assessed with four fern and four moss species in an experimental setup in a climate chamber. The experiment showed significant negative, though varied effects of elevated climbing chalk concentrations on the germination and survival of both ferns and mosses. The study thus suggests that along climbing routes, elevated climbing chalk concentration can occur even were no chalk traces are visible and that climbing chalk can have negative impacts on rock‐dwelling organisms.

## Graphical abstract

Rock climbing is popular all around the globe and so is the use of climbing chalk (magnesium carbonate hydroxide powder that is applied to enhance grip friction). However, the impact of climbing chalk on rock‐dwelling organisms has not been properly studied so far. The article presents a first assessment of the effects of climbing chalk. We found distinctively elevated climbing chalk concentrations along climbing routes and pronounced effects of climbing chalk on the germination and survival of ferns and mosses in an experiment.

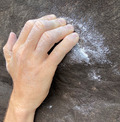

## 1 INTRODUCTION

The number of climbers is rising worldwide, since the popularity of rock climbing, as a sport activity, is continuously increasing. Its Olympic debut in Tokyo 2021 will probably further accentuate this trend (Attarian & Keith, 2008; IOC, 2016). Along with this rise of rock climbing, previously uninfluenced rock habitats are increasingly frequented by humans, and their adverse impacts require mitigation (Hanemann, 1999; Holzschuh, 2016). Several authors have studied the impact of climbing on multiple organismic groups (reviewed in Holzschuh, 2016) and predominantly reported negative effects. However, Holzschuh (2016) pointed out that most of the studies on the impact of climbing are based on the direct comparison between climbed and unclimbed rocks (e.g., Müller, Rusterholz, & Baur, 2004; Nuzzo, 1996; Rusterholz, Müller, & Baur, 2004) and thereby might overestimate the impact of climbing because of confounded abiotic differences between climbed and unclimbed rocks such as terrain roughness. Nevertheless, recent studies accounting for this methodological drawback still found negative effects of climbing activities on rock vegetation (March‐Salas et al., 2018; Tessler & Clark, 2016) and showed that increasing climbing intensity goes along with increasing alterations of species communities on rocks (Lorite et al., 2017; Schmera, Rusterholz, Baur, & Baur, 2018). While for rock‐nesting bird species, simple human presence leads to disturbance (Camp & Knight, 1998; Covy, Benedict, & Keeley, 2019), negative effects of rock climbing on sessile rock‐dwelling organisms are mainly attributed to mechanical disturbances such as trampling and removal of soil and vegetation (Holzschuh, 2016). However, climbing chalk—a component unique to climbing among all outdoor activities—was merely considered as a visual indicator for climbing activities on rocks so far (Camp & Knight, 1998; Thiel & Spribille, 2007; Adams & Zaniewski, 2012; Clark & Hessl, 2015), while its potential chemical impact on rock‐dwelling species has rarely been mentioned (Holzschuh, 2016; Tessler & Clark, 2016). In fact, the only published study on the potential chemical impact of climbing chalk was conducted by Fickert (2014), who investigated the potential increase in soil pH due to climbing chalk at the base of climbed boulders and found no difference between soil pH at the base of climbed and unclimbed boulders. Given the scarcity of information, the chemical impact of climbing chalk on rock‐dwelling vegetation remains largely unknown,

Climbing chalk is a fluffy white powder that consists of magnesium carbonate hydroxide (1‐4MgCO_3_·Mg(OH)_2_·3‐5H_2_O), which is also known as magnesia alba or basic magnesium carbonate (Ropp, 2013; Shand, 2006). It dries hand sweat and thereby enhances grip friction. Traditionally used in gymnastics, in the 1950s it was introduced to bouldering, which is a low height (generally < 4 m, often on boulders), ropeless subdiscipline of climbing (Gill, 1969; Niegl, 2009; Tessler & Clark, 2016). Nowadays, climbing chalk is perceived as an inherent component of all kinds of rock climbing, but its use is probably most extensive in bouldering (Attarian & Keith, 2008; Niegl, 2009). Magnesium carbonate hydroxide is barely soluble in pure water, and in aqueous suspension, it has a pH of around 10.5, its solubility is better if water contains CO_2_, and it readily dissolves in diluted acid (Budavari, O’Neil, Smith, Heckelman, & Kinneary, 1996; Ropp, 2013; Shand, 2006). With regard to the pH dependency of plant nutrient uptake and the vital role of magnesium as macronutrient, climbing chalk can be expected to impact plant growth (Barker & Pilbeam, 2015). This potential impact should be interdependent with rock chemistry, as acidic conditions on siliceous rock (e.g., granite or gneiss) strongly contrast the alkaline properties of climbing chalk, while alkaline conditions on carbonatic rock (e.g., limestone or dolomite) are more in line with the chemical properties of climbing chalk (Kinzel, 1983). Hence, calcifuge species might be more susceptible to climbing chalk than calcicoles. Among the diverse life forms of rock‐dwelling plants, ferns and mosses—common life forms in most cliff ecosystems (Larson, Matthes, & Kelly, 2000)—should be particularly sensitive to climbing chalk as their early gametophytic stages (prothallia and protonema) lack regulatory mechanisms and directly absorb water with their single‐cell‐layer plant bodies (Jahns, 1983).

In the present study, we addressed two fundamental aspects contributing to the understanding of the potential impact of climbing chalk on rock‐dwelling ferns and mosses. First, in order to gain information on the extent of climbing chalk present along climbing routes, we measured its presence, concentrations, and distribution on gneiss boulders used for bouldering. Second, in order to explore a potential chemical effect of climbing chalk on rock‐dwelling ferns and mosses, we experimentally tested whether different climbing chalk concentrations affected the germination and survival of rock‐dwelling ferns and mosses in a climate chamber experiment.

## 2 MATERIAL AND METHODS

### 2.1 Study site and measurements of climbing chalk on climbed boulders

**Figure 1:**
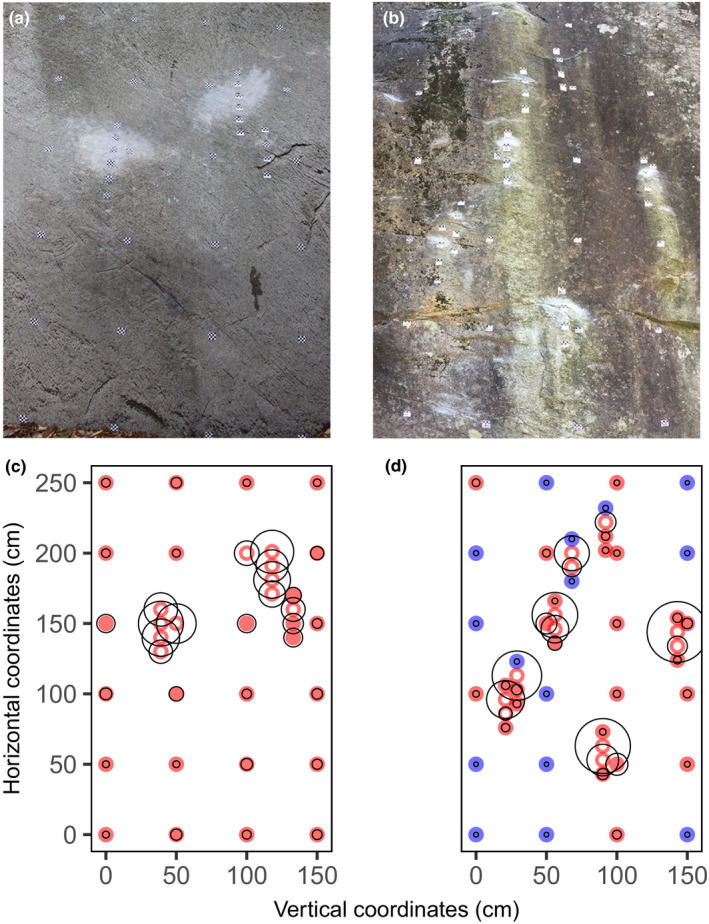
Example of climbing chalk (magnesium) distribution on two climbed boulders (boulders B and E, Table 1). On the photographs (a, b) of the assessed 2.5 m × 1.5 m rectangles stickers mark sampling points, and white climbing chalk traces are visible predominantly around the climbing holds. The corresponding graphics (c, d) visualize the amount of climbing chalk (magnesium) measured on the sampling points. Red: elevated values above threshold level; blue: values below threshold level; white dots: visible climbing chalk traces at sampling points; area within black circles: proportional amount of climbing chalk measured at sampling points.

In order to obtain first information on the presence, distribution, and concentration of climbing chalk on climbed rock, we used the following sampling scheme. We sampled bouldering routes due to their accessibility (as compared to roped climbing routes) on siliceous boulders that enabled swab‐sampling with diluted acid, which thereby dissolves climbing chalk traces potentially present on the rock but not the rock itself (compared to limestone). We selected nonoverhanging rock surfaces with flat topographies of similar slope and equal size in order to increase comparability (Figures 1, S1).

Field sampling took place in southern Switzerland (Ticino) in two well‐known bouldering areas Cresciano and Chironico, which both comprise > 1,000 bouldering routes (Ambrosio, Cameroni, Grizzi, Lodi, & Vonarburg, 2006a, 2006b). Both bouldering areas are holocene landslide deposits of gneiss boulders of the Penninic Leventina nappe whose granitic orthogneisses generally have a low magnesium content (Claude et al., 2014; Rütti, Marquer, & Thompson, 2008). In each bouldering area, we sampled the first three rock faces we came across that were not markedly overhanging (i.e., slope < 95°) and had a rather flat topography on a surface of 2.5 m in height and 1.5 m in width enclosing a substantial part of a bouldering route (Table 1). Within the 2.5 m × 1.5 m area (Figure 1), on each boulder, two sets of sampling points were collected. (a) The general distribution of climbing chalk along the bouldering route was sampled on a 0.5 m raster grid, and (b) the climbing holds were targeted by sampling points on the centers of climbing holds as well as vertically 10 cm above, 10 cm below, and 20 cm below the climbing hold. As a control, we additionally sampled a total of 20 sampling points on unclimbed boulders (10 points per bouldering area). For each sampling point, we inspected an area of 2 cm × 1 cm, noted whether climbing chalk deposits were visible or not and carefully tabbed off the 2 cm × 1 cm area with a medical swab (FLOQSwabs 502CS01, Copan, Brescia, Italy) that was slightly wetted in 2% nitric acid in order to dissolve and take up a maximum amount of climbing chalk potentially present on the sampling point. The swabs were put into individual 15‐ml centrifuge tubes containing 10 ml pure water. In the laboratory, magnesium content was determined with ICP‐OES (Inductively Coupled Plasma‐ Optical Emission Spectrometry; Optima 7300 DV, Perkin Elmer, Waltham, USA). As a conservative threshold level for elevated climbing chalk concentrations, we defined the empirical 99.7nd percentile (mean plus three standard deviations) of the magnesium concentrations measured on the 20 control sampling points on unclimbed boulders (i.e., 0.00126 mg/cm^2^ magnesium).

**Table 1:** Sampled bouldering routes in the two bouldering areas Cresciano and Chironico

ID	Climbing grade*	Aspect	Inclination	Coordinates	Sampling date
Cresciano					
A	6a	N	Vertical (~90°)	46.28573/9.00702	22.10.2016
B	6b+	SW	Inclined (<90°)	46.28695/9.00664	22.10.2016
C	6b	S	Vertical (~90°)	46.28584/9.00689	13.10.2017
Chironico					
D	6a+	SW	Vertical (~90°)	46.430220/8.846502	13.10.2017
E	NA	SO	Inclined (<90°)	46.427499/8.849307	13.10.2017
F	7c+	W	Vertical (~90°)	46.430650/8.848645	14.10.2017

Note

Climbing grade (Fontainebleau‐Scale), aspect and inclination of the sampled 2.5 m × 1.5 m areas, coordinates (latitude/longitude WGS84), and sampling date.

* Ambrosio et al. (2006a, 2006b).

### 2.2 Germination and survival experiment

In order to explore the potential impact of climbing chalk on rock‐dwelling fern and moss species under controlled conditions, we set up a factorial experiment in which we sowed spores on agar plates with different climbing chalk concentrations and assessed their germination and survival. Spores of four rock‐dwelling fern species (*Asplenium septentrionale, A. trichomanes* ssp. *quadrivalens*, *Cystopteris fragilis,* and *Polypodium vulgare*) and four rock‐dwelling moss species (*Grimmia pulvinata*, *Hedwigia ciliata*, *Hypnum cupressiforme,* and *Orthotrichum anomalum*) covering a broad spectrum of rock habitat types were collected in Switzerland (Table 2). At each site, several fertile fern fronds with freshly opened sporangia or moss sporophytes from about five different individuals were pooled, dried, and stored at room temperature. Fern spores were purified using different sieves (1 cm–50 µm), and moss spores were purified by separating sporophyte debris from spores using forceps.

**Table 2:** Rock‐dwelling ferns and mosses used in the germination and survival experiment

Species	Habitat preferences*	Accession	Run
Ferns:			
*Asplenium septentrionale* (L.) Hoffm.	Exposed siliceous rocks; calcifuge	Ausserberg (VS), 46.31463/7.84271 1.7.2014	2017
*Asplenium trichomanes* subsp. *quadrivalens* D. E. Mey.	Calcareous rocks and mortared walls; calcicole	Wädenswil (ZH) 47.22331/8.67640 12.10.2012	2018
*Cystopteris fragilis* (L.) Bernh.	Shady basic rocks and walls; calcicole	Wädenswil (ZH) 47.22288/8.67689 12.7.2018	2018
*Polypodium vulgare* L.	Acidic rocks, also epiphytic; calcifuge	Arth‐Goldau (SZ) 47.04790/8.55601 16.9.2017	2018
Mosses:			
*Grimmia pulvinata* (Hedw.) Sm.	Calcareous rocks, walls and concrete; calcicole	Wädenswil (ZH) 47.22131/8.67664 4.5.2017	2018
*Hedwigia ciliata* (Hedw.) P.Beauv.	Exposed siliceous rocks; calcifuge	Bellinzona (TI) 23.10.2016 46.18851/9.03073	2017
*Hypnum cupressiforme* Hedw.	Siliceous rocks, rotting wood, trees and soil; calcifuge	Wädenswil (ZH) 47.21805/8.67905 2.3.2017	2017
*Orthotrichum anomalum* Hedw.	Calcareous rocks, walls and concrete; calcicole	Wädenswil (ZH) 47.22151/8.67691 4.5.2017	2017

Note

Typical habitat and pH preferences (calcicole or calcifuge), site (locality, latitude/longitude WGS84), and collection date of accession and year in which the experiment was conducted (run).

* Ellenberg et al. (1992); Jahns (1983); Lauber, Wagner, and Gygax (2018)

The experiment was conducted in 35 mm diameter petri dishes each containing 4 ml of 0.45% agar medium (A 7002 Sigma‐Aldrich, St. Louis, USA). We prepared four different types of agar media that varied in their climbing chalk concentrations: The 0% climbing chalk medium (control) was based on pure water, the 100% climbing chalk medium based on pure water saturated with climbing chalk (26 mg/L magnesium; Loose White Gold Chalk, Black Diamond, Innsbruck, Austria), and the 50% and 25% media based on 1:1 and 1:3 dilutions of the saturated climbing chalk solution. Because of the nutrient poverty of rock habitats (Larson et al., 2000) and because agar contains some nutrients (Bridson & Brecker, 1970), no additional nutrients were added.

We did not sterilize spores to avoid unwanted influences due to the sterilization process (Camloh, 1999). Therefore, spore batches were first checked for contamination with fungi etc. by sowing ten petri dishes per spore batch. Batches that showed contaminations after one week on more than three dishes were discarded. For sowing, a pinch of spores was suspended in 500 µl pure water containing 0.05% of the nonionic detergent Tween 80 (Sigma‐Aldrich, Louis, USA), which prevented spores from clumping. After soaking for two hours, spore suspensions were diluted to a concentration of 10–15 spores/µl. One droplet of 2 µl spore suspension was placed into the petri dish with a piston pipette resulting in ca. 20–30 spores dispersed in a circle of 4 mm diameter. Petri dishes were sealed with parafilm.

In the experiment, every species–concentration combination was replicated in twelve petri dishes (i.e., 384 dishes in total). Germinated and living plants were counted under a stereo microscope weekly for 6 weeks. The number of sown spores was determined during the first counting. *Hypnum cupressiforme* (Table 2) was counted twice a week during 2.5 weeks only, because later on, it was impossible to distinguish individuals due to its filamentous and fast growing protonema (Figure S2).

The experiment was carried out in two runs (due to limited availability of climate chamber; 02.05.‐11.07.2017 and 18.10.‐04.12.2018, respectively; Table 2) in the same four shelf climate chamber (RUMED 1301) at a constant temperature of 22°C, a relative humidity of 80%, and a 16/8‐hr day/night cycle (eight fluorescent tubes; Philips 58W, TLD480 REFLEX, each about 5,240 lumens). The 48 petri dishes per species were equally distributed among four trays of acrylic glass, each tray containing three randomly assigned replicates of each of the four climbing chalk concentrations. Each of the four trays was randomly assigned to one of the four shelves (one tray per species per shelf). A potential temperature gradient among shelves was accounted for by weekly moving the bottom shelf to the top and the other shelves one position downwards. Tray positions on shelves were randomized weekly and dish position per tray during counting.

From the six countings per petri dish, we derived two response variables: (a) germination rate being the maximum number of plants observed at any counting event divided by the number of sown spores and (b) survival rate being the number of surviving plants at end of the experiment divided by the maximum number of plants observed at any counting event.

The experiment corresponded to a split plot design (Altman & Krzywinski, 2015), and we thus analyzed the germination and survival rates which based on count data of binominal nature with generalized linear mixed models using the binomial family with the logit link function (package lme4; Bates, Mächler, Bolker, & Walker, 2015) in R (R Core Team, 2017). As fixed effects, we considered the block effect shelf (eight levels: four climate chamber shelfs times two runs), the treatment effect species (eight levels, one per species), the treatment effect climbing chalk concentration (four levels: 0%, 25%, 50%, 100%), and the interaction between species and climbing chalk concentration. As random effects, we included the further factors given by the experimental design: the two runs (i.e., the two time periods in which the experiment was conducted; see above), the 32 plexiglass trays on which the petri dishes were placed (four trays per species, each tray corresponding to a plot), and, in order to accommodate for overdispersion, individual petri dishes (corresponding to split plots). The fixed effects were tested in sequential likelihood ratio tests (deviance tables; Nelder & Wedderburn, 1972), and the differences of adjacent factor levels of climbing chalk concentrations were tested with forward difference contrasts (Venables & Ripley, 2002).

## 3 RESULTS

### 3.1 Measurements of climbing chalk on climbed boulders

**Figure 2:**
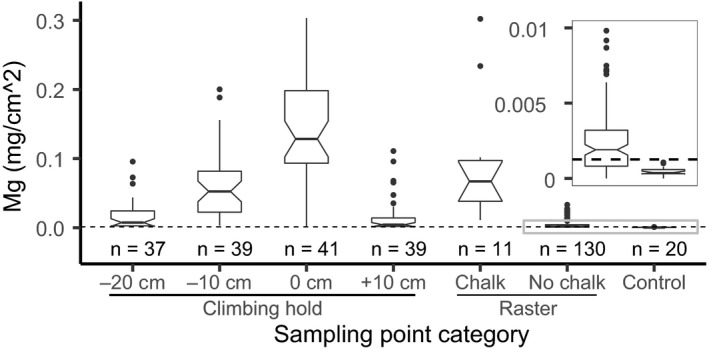
Amount of climbing chalk (magnesium) measured on climbed and unclimbed (control) boulders. Sampling points at and around climbing holds are grouped by their vertical distances to the climbing hold (−20 cm, −10 cm, 0 cm, +10 cm), raster sampling points by visibility of climbing chalk traces (chalk, no chalk). The dotted line indicates the threshold above which climbing chalk measurements were considered as elevated. The inset shows the content of the gray box at an enlarged scale.

The measurements on climbed boulders revealed distinctively elevated climbing chalk (magnesium) concentrations, with highest amounts on climbing holds and raster points with visible climbing chalk traces (Figure 1, Figure S1). At and around the totally 41 climbing holds, climbing chalk visbility and measured concentrations were highest at the middle of holds, followed by the sampling points 10 cm below, 20 cm below, and 10 cm above the climbing holds (Figure 2; Table 3). The sampling of totally 141 raster points revealed that 85 (65%) of the 130 sampling points without any visible climbing chalk traces showed elevated climbing chalk concentrations above the defined threshold level of 0.00126 mg/cm^2^ magnesium (Table 3, Figure 2). One of the six surveyed boulders exceeded the threshold level of climbing chalk concentration at every sampling point (Figure 1c).

**Table 3:** Summary of the total 317 sampling points assessed on gneiss boulders

	*N*	Climbing chalk visible %	Magnesium elevated %
Climbing hold	156	57	95
+10 cm	39	13	82
0 cm	41	100	100
−10 cm	39	79	100
−20 cm	37	32	97
Raster	141	8	67
Chalk	11	100	100
No chalk	130	0	65
Control	20	0	0

Note

Percentages of sampling points with visible climbing chalk traces and elevated climbing chalk concentrations (magnesium) for each of the different types of sampling points: on and above and below climbing holds (+10 cm, 0 cm, −10 cm, −20 cm), on raster points with and without visible traces of climbing chalk and on unclimbed boulders (control).

### 3.2 Germination and survival experiment

**Figure 3:**
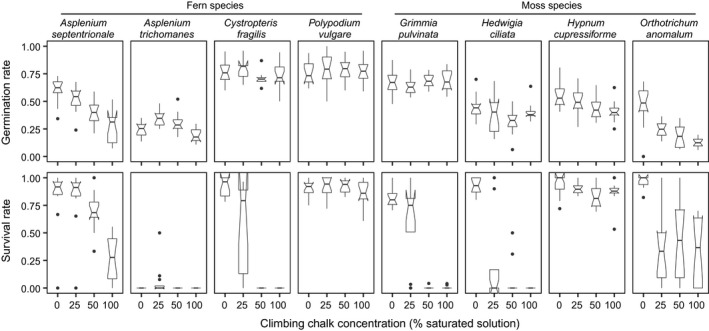
Germination and survival rates of four fern and four moss species on agar with four climbing chalk concentrations.

Response patterns to climbing chalk differed among species (Figure 3): Germination rate and survival rate of *Asplenium septentrionale* declined with rising climbing chalk concentrations, *A. trichomanes* showed generally low germination and almost no survival across all media, *Cystopteris fragilis* germinated equally well under all climbing chalk concentrations, while survival was obviously reduced on media containing climbing chalk, and the response of *Polypodium vulgare* in both germination and survival was rather uniform across climbing chalk concentrations. The moss species showed no obvious systematic difference to the fern species but also rather diverse response patterns (Figure 3): *Grimmia pulvinata* and *Hedwigia ciliata* germinated equally well on all media, while survival was reduced on media containing climbing chalk, germination and survival rates of *Hypnum cupressiforme* showed a slight decline with rising climbing chalk concentrations, and *Orthotrichum anomalum* showed lower germination and survival on media containing climbing chalk. Noteworthy, the plants of all species appeared less vigorous on climbing chalk media (examples in Figure S2).

The effects of species, climbing chalk concentration, and their interaction were significant on germination and survival rates, while the block effect shelf had no significant influence (Table 4). The effects of rising climbing chalk levels were negative: Between the 25% and 50% media as well as between the 50% and 100% media, germination and survival rates decreased significantly, while the differences between 0% and 25% were not significant (Table 5).

**Table 4:** Analysis of deviance of fixed effects, sequentially added to the random effect model, on the germination and survival rates of fern and moss species

Fixed effect	*df* model	Deviance model	χ^2^	*df*	*p*
Germination rate					
Intercept	4	2,091	‐	‐	‐
Shelf	11	2,085	6.2	7	.52
Species	17	1,981	103.9	6	<.0001
Concentration	20	1,922	58.9	3	<.0001
Species: concentration	41	1,797	125.2	21	<.0001
Survival rate					
Intercept	4	1,513	‐	‐	‐
Shelf	11	1,508	4.6	7	.71
Species	17	1,408	99.8	6	<.0001
Concentration	20	1,244	164.4	3	<.0001
Species: concentration	41	1,024	220.2	21	<.0001

**Table 5:** Coefficients obtained by forward difference contrasts of the four levels of climbing chalk concentration in the full generalized linear mixed models for germination and survival rates of all four fern and four moss species studied together

Contrast	Estimate	Std. Error	Z‐value	*p*
Germination rate				
25% versus 0%	−0.300	0.176	−1.703	.088
50% versus 25%	−0.570	0.177	−3.224	.001
100% versus 50%	−0.516	0.188	−2.750	.006
Survival rate				
25% versus 0%	−0.079	0.598	−0.132	.895
50% versus 25%	−1.542	0.570	−2.707	.007
100% versus 50%	−2.044	0.564	−3.624	.0002

## 4 DISCUSSION

Our study suggests that along climbing routes, elevated climbing chalk concentration can occur even were no chalk traces are visible and that climbing chalk can have negative impacts on the germination and early survival of rock‐dwelling ferns and mosses.

### 4.1 Measurements of climbing chalk concentrations on climbed boulders

To our knowledge, this is the first study presenting measurements of climbing chalk (magnesium) on climbed rock. We deduced climbing chalk input by climbers by measuring the amount of magnesium on climbed boulders, which we set in relation to the amount of naturally occurring magnesium detected on unclimbed boulders of the same rock type. In the samples taken in the middle of climbing holds, vertically 10 cm above, 10 cm below, and 20 cm below, the highest concentrations were measured in the middle of the climbing holds where climbing chalk traces were always visible (Figure 2, Table 3). Above climbing holds, both visible climbing chalk traces and concentrations diminished promptly. In contrast, below climbing holds, visible climbing chalk traces, and climbing chalk concentrations diminished gradually. This trend of primarily downward dispersal of climbing chalk on rock faces was also noticeable in raster sampling points without visible climbing chalk traces, but often elevated climbing chalk concentrations (Figure 1d, Figure S1). We hypothesize three interdependent main factors influencing the distribution of climbing chalk on climbed rocks: (a) Climbing intensity (Schmera et al., 2018) should be positively correlated with the input of climbing chalk. (b) The climbing routes' microtopography (including slope; Kuntz & Larson, 2006) will affect the amount and frequency by which a climber applies climbing chalk and also the way how climbing chalk is dispersed. (c) The dispersion pathway—either leaching with runoff rainwater or as dust during climbing (Weinbruch, Dirsch, Ebert, Hofmann, & Kandler, 2008) or brushing/cleaning of climbing holds (Niegl, 2009)—contributes to the spatial extent by which climbing chalk spreads on a rock away from climbing routes and holds. The examples given in Figure 1 illustrate these aspects. The popular route called “the never ending story” (Figure 1a; Ambrosio et al., 2006b) exceeded the threshold level of climbing chalk concentration at every sampling point (Figure 1c). It is climbed intensively, its microtopography necessitates two extensive holds, and runoff rainwater and dust from climbing and brushing of holds further spread climbing chalk on the <90° inclined rock. In contrast, on a less popular, unnamed route (Figure 1b; Ambrosio et al., 2006a), elevated climbing chalk concentrations were more confined to climbing holds and adjacent areas below them (Figure 1d). The route is only rarely climbed, its climbing holds are small, and there were no signs of brushing activities. Here, runoff rainwater on the <90° inclined rock might not only distribute climbing chalk but also cause magnesium to leach and levels to decrease over time.

### 4.2 Germination and survival experiment

We are not aware of other studies on the effect of climbing chalk on mosses and ferns or on any other rock‐dwelling organisms. Methodologically, we approached this question with a germination experiment with different climbing chalk concentrations on agar in a climate chamber. On the one hand, conditions on agar plates in a climate chamber are far from the environmental conditions on rock habitats, but on the other hand, the chosen approach allowed varying climbing chalk concentrations in a controlled way and for quantitatively measuring germination and survival. While standard ecotoxicological germination tests assess germination rate as the fraction of germinated seeds after a defined time span (OECD, 2006; Wang & Keturi, 1990), we defined germination rate as the ratio between the maximum number of germinated and living plants and the number of spores sown per petri dish. This was necessary because of the different germination behavior of the assessed species. For instance, *Hypunum cupressiforme* had already started germinating three days after sowing, and after only one week, 83% of its petri dishes with spores from this species reached their maximum numbers of living plants. In contrast, spores of *Orthotrichum anomalum* took two weeks to start germination (Figure S3). Across all species, except *H. cupressiforme*, after four weeks 87% of the petri dishes had reached their maximum numbers of living plants. Hence, the germination process in this study can be considered as rather complete. However, survival rate—the fraction of plants surviving until the end of the experiment—reflected an ongoing process. This becomes obvious when considering the development of the studied species over time (Figure S3). While for *Asplenium trichomanes,* nearly all individuals had died after six weeks, the number of living plants per petri dish of most other species was steadily decreasing and more accentuated in higher climbing chalk concentrations.

Germination and survival rates differed among species (Figure 3; Table 4), but there was a lack of correspondence between species' ecology (Table 2) and their response to elevated climbing chalk concentration in the experiment (Figure 3). One would expect calcicoles to be more tolerant to higher pH values and magnesium concentrations, because they should be adapted to high pH levels and high ion concentrations of calcium, which is chemically similar to magnesium (Barker & Pilbeam, 2015; Lee, 1999). Nevertheless, the calcicoles *Cystopteris fragilis* and *Grimmia pulvinata* showed a similar response as the calcifuge *Hedwigia ciliata* with almost no survival on 50% and 100% climbing chalk media, whereas the calcifuge *A. septentrionale* responded with a gradual decline in germination and survival, and the calcicole *O. anomalum* exhibited a pattern inbetween. The calcicole subspecies of *A. trichomanes* exhibited generally low germination and survival at all climbing chalk concentrations, a result that could also have been caused by rather old spores of this species used in the experiment (Table 2; Camloh, 1999). Only *Polypodium vulgare* and *H. cupressiforme* showed congruence in their response patterns (only slight differences in germination and survival among different climbing chalk) with their ecology (weakly calcifuge and also growing epiphytic). Summing up, a simple calcicole–calcifuge classification (Lee, 1999) did not predict the species' response to elevated climbing chalk concentrations found in the present study. The observed differences rather reflect species‐specific reactions to pH and ion concentrations as documented for diverse fern and moss species (Suo, Chen, Zhao, Shi, & Dai, 2015; Wiklund & Rydin, 2004).

### 4.3 Synthesis and outlook

On six climbing routes on gneiss used for bouldering, on regularly spaced raster points, elevated climbing chalk levels were measured even at 65% of the sampling points without any visual traces of climbing chalk (Table 3). In the laboratory experiment with four fern and four moss species, increasing climbing chalk concentrations impaired germination and survival (Table 5). These two results may be set into relation through the amount of climbing chalk involved. For instance, the amount magnesium present at the threshold level of elevated climbing chalk concentrations (0.00126 mg/cm^2^) is equivalent to a ca. 1 mm thick layer of 50% saturated climbing chalk medium, which significantly impaired germination and survival in the experiment (13 mg/L). One could thus conclude that even on 65% of the raster sampling points without any visual traces of climbing chalk, rock‐dwelling plant species (Table 2) may well be negatively affected. However, unlike thawing salt, which simply increases the concentration of sodium (Na^+^) and chloride (Cl^−^) ions (Blomqvist, 1998), the situation with climbing chalk is more complex. Solubility of climbing chalk positively depends on water acidity (Budavari et al., 1996), which is influenced by the acidity of rain and the runoff through substrates and on rock (Larson et al., 2000). When dissolved, the ions involved in magnesium carbonate hydroxide are magnesium cations (Mg^2+^), which increase the ion concentration, and the anions carbonate (CO_3_^2−^) and hydroxide (OH^−^), which additionally increase pH. In solution, the anionic components of climbing chalk are in acid‐base equilibria: hydroxide with water (H_2_O) and carbonate with bicarbonate (HCO_3_^−^) and carbonic acid (H_2_CO_3_), which is in solution equilibrium with CO_2_ in the air. Hence, depending on solution and acid‐base equilibria, climbing chalk can evolve into other compounds than the initial magnesium carbonate hydroxide. This interplay of water acidity and solubility of climbing chalk should be substantially influenced by rock type. Acidic conditions on siliceous rock, like the gneiss rock on which we conducted our measurements, enhance solubility of climbing chalk and thereby may also enhance dislocation of magnesium with runoff water. In contrast, alkaline conditions on limestone lower the solubility of climbing chalk, which may thereby enhance its persistence on rock and dislocation with runoff water may be predominantly in form of climbing chalk particles, rather than in its dissolved ions. Further, on porous sandstone persistence of climbing chalk is reported to be particularly enhanced as it irreversibly stains and alters sandstone surfaces (Attarian & Keith, 2008; Niegl, 2009; Huddart & Stott, 2019) In addition, rock type is also a main determinant for plant community composition on rocks (Spitale & Nascimbene, 2012). Calcicole and calcifuge communities might react differently to climbing chalk rather due to the different chemical behavior of climbing chalk on different rock types than due to calcicole–calcifuge adaptations of plant species. For the latter, we found no evidence in our experiment.

In the results of the diverse studies that documented impacts of climbing on rock vegetation (Lorite et al., 2017; March‐Salas et al., 2018; Müller et al., 2004; Nuzzo, 1996; Rusterholz et al., 2004; Schmera et al., 2018; Tessler & Clark, 2016), the potential chemical impact due to climbing chalk and the mechanical impacts of climbing such as trampling and removal of soil and vegetation are usually confounded on climbed rocks (Holzschuh, 2016). In contrast, our study assessed the distribution of climbing chalk separately from its impact on species in an experiment, but the real impact of climbing chalk on rock‐dwelling plants under natural conditions is difficult to deduce. To this end, studies in climbing areas that only allow climbing chalk on a subset of their routes (Heinicke, 2001; Werdermann, 1993) might allow to compare the impact of climbing with and without climbing chalk. Furthermore, it is worthwhile to continue with an experimental approach by applying climbing chalk on unclimbed rock and study its in situ impact on rock‐dwelling species (besides bryophytes and ferns, also including lichens and flowering plants) on different rock types. Finally, recent advances in drone and imaging technology may allow for mapping and analyzing visible climbing chalk traces and vegetation health along climbing routes with hyperspectral imaging by means of unmanned aerial vehicles (Peng et al., 2020; Strumia, Buonanno, Aronne, Santo, & Santangelo, 2020; Zhang, Zhang, Wei, Wang, & Huang, 2020).

### 4.4 Recommendations for conservation practice

Our study indicates that climbing chalk might negatively affect mosses and ferns growing along climbing routes. Although there is clear necessity for further research on this issue, we precautionary suggest taking into account the potential impact of climbing chalk when developing conservation measures for rare rock‐dwelling species in close vicinity of climbing routes (e.g., when erratic boulders that harbor rare species are used for bouldering; Hepenstrick, Urmi, Meier, & Bergamini, 2016; Mazenauer, Holderegger, Krüsi, & Hepenstrick, 2014). In order to judge upon a potential threat by climbing chalk, it helps to consider climbing intensity, microtopography, and dispersion pathways (leaching with runoff rainwater and dust from climbing or brushing). In the experiment with different climbing chalk concentrations, negative impacts of climbing chalk were present in calcicole and calcifuge species. Hence, potential impacts of climbing chalk are not necessarily restricted to calcifuge plant species. Furthermore, alternatives to climbing chalk such as adhesive colophony resin or absorptive balls which absorb excess moisture from hands (Niegl, 2009) might be assessed as a potential remedy in cases where the use of climbing chalk is problematic.

## CONFLICT OF INTEREST

The authors declare no conflict of interest.

## AUTHOR CONTRIBUTIONS

DH, AB, and RH: Design. DH: Material preparation; data collection; and analysis. DH: Writing manuscript. AB and RH: Editorial advice. All authors read and approved the final manuscript.

## Supporting information

FigS1

FigS2

FigS3

## Data Availability

The datasets generated and analyzed in this study are available on Dryad: https://doi.org/10.5061/dryad.cc2fqz649

## References

[ece36773-bib-0001] Adams, M. D. , & Zaniewski, K. (2012). Effects of recreational rock climbing and environmental variation on a sandstone cliff‐face lichen community. Botany, 90, 253–259. 10.1139/b11-109

[ece36773-bib-0002] Altman, N. , & Krzywinski, M. (2015). Split plot design. Nature Methods, 12, 165–166. 10.1038/nmeth.3293 25879095

[ece36773-bib-0003] Ambrosio, A. , Cameroni, C. , Grizzi, R. , Lodi, R. , & Vonarburg, N. (2006a). Chironico boulder. Bellinzona: Ticino Boulder.

[ece36773-bib-0004] Ambrosio, A. , Cameroni, C. , Grizzi, R. , Lodi, R. , & Vonarburg, N. (2006b). Cresciano boulder. Bellinzona: Ticino Boulder.

[ece36773-bib-0005] Attarian, A. , & Keith, J. (2008). Climbing management, a guide to climbing issues and the development of a climbing management plan. Boulder: The Access Fund.

[ece36773-bib-0006] Barker, A. V. , & Pilbeam, D. J. (2015). Handbook of plant nutrition. Boca Raton: CRC Press.

[ece36773-bib-0007] Bates, D. , Mächler, M. , Bolker, B. , & Walker, S. (2015). Fitting linear mixed‐effects models using lme4. Journal of Statistical Software, 67, 1–48. 10.18637/jss.v067.i01

[ece36773-bib-0008] Blomqvist, G. (1998). Impact of de‐icing salt on roadside vegetation: A literature review. Linköping: Swedish National Road and Transport Research Institute.

[ece36773-bib-0009] Bridson, E. Y. , & Brecker, A. (1970). Design and formulation of microbial culture media In Norris J. R., & Ribbons D. W. (Eds.), Methods in microbiology 3A (pp. 229–295). London: Cambridge University Press.

[ece36773-bib-0010] Budavari, S. , O’Neil, M. J. , Smith, A. , Heckelman, P. E. , & Kinneary, J. F. (Eds.) (1996). The Merck index, an encyclopedia of chemicals, drugs and biologicals. Whitehouse Station: Merck.

[ece36773-bib-0011] Camloh, M. (1999). Spore age and sterilization affects germination and early gametophyte development of *Platycerium bifurcatum* . American Fern Journal, 89, 124–132. 10.2307/1547346

[ece36773-bib-0012] Camp, R. J. , & Knight, R. L. (1998). Rock climbing and cliff bird communities at Joshua Tree National Park, California. Wildlife Society Bulletin, 26, 892–898. https://jstor.org/stable/3783567

[ece36773-bib-0013] Clark, P. , & Hessl, A. (2015). The effects of rock climbing on cliff‐face vegetation. Applied Vegetation Science, 18, 705–715. 10.1111/avsc.12172

[ece36773-bib-0014] Claude, A. , Ivy‐Ochs, S. , Kober, F. , Antognini, M. , Salcher, B. , & Kubik, P. W. (2014). The Chironico landslide (Valle Leventina, southern Swiss Alps): Age and evolution. Swiss Journal of Geosciences, 107, 273–291. 10.1007/s00015-014-0170-z

[ece36773-bib-0015] Covy, N. , Benedict, L. , & Keeley, W. H. (2019). Rock climbing activity and physical habitat attributes impact avian community diversity in cliff environments. PLoS One, 14, e0209557 10.1371/journal.pone.0209557 30650086 PMC6334907

[ece36773-bib-0016] Ellenberg, H. , Weber, H. E. , Düll, R. , Wirth, V. , Werner, W. , & Paulissen, D. (1992) Zeigerwerte von Pflanzen in Mitteleuropa. Göttingen: Goltze.

[ece36773-bib-0017] Fickert, T. (2014). Zum Einfluss des Klettersports auf silikatische Felsökosysteme. Mitteilungen der Fränkischen Geographischen Gesellschaf, 59, 47–58. http://fgg‐erlangen.de/fgg/ojs/index.php/mfgg/article/view/262

[ece36773-bib-0018] Gill, J. (1969). The art of bouldering. American Alpine Journal, 16, 355–357.

[ece36773-bib-0019] Hanemann, B. (1999). The sustainable management of climbing areas in Europe. Gland: IUCN.

[ece36773-bib-0020] Heinicke, D. , Friedrich, J. , Glaser, C. , Heinicke, F. , Scharnweber, M. , & Seifert, F. (2001). Kletterführer Sächsische Schweiz, Gebiet der Steine. Dresden: Berg‐ und Naturverlag.

[ece36773-bib-0021] Hepenstrick, D. , Urmi, E. , Meier, M. K. , & Bergamini, A. (2016). Die Moosflora des silikatischen Findlings Alexanderstein in Küsnacht (ZH). Meylania, 57, 15–23. 10.21256/zhaw-2029

[ece36773-bib-0022] Holzschuh, A. (2016). Does rock climbing threaten cliff biodiversity? A critical review. Biological Conservation, 204, 153–162. 10.1016/j.biocon.2016.10.010

[ece36773-bib-0023] IOC (2016). IOC approves five new sports for Olympic Games Tokyo 2020. https://www.olympic.org/news/ioc‐approves‐five‐new‐sports‐for‐olympic‐games‐tokyo‐2020 Accessed 22 November 2019.

[ece36773-bib-0024] Jahns, H. M. (1983). Collins guide to the ferns, mosses, and lichens of Britain and North and Central Europe. London: Collins.

[ece36773-bib-0025] Kinzel, H. (1983). Influence of limestone, silicates and soil pH on vegetation In Lange O. L., Nobel P. S., Osmond C. B., & Ziegler H. (Eds.), Encyclopedia of plant physiology, physiological plant ecology III (12) (pp. 201–244). Berlin: Springer.

[ece36773-bib-0026] Kuntz, K. L. , & Larson, D. W. (2006). Influences of microhabitat constraints and rock‐climbing disturbance on cliff‐face vegetation communities. Conservation Biology, 20, 821–832. 10.1111/j.1523-1739.2006.00367.x 16909575

[ece36773-bib-0027] Larson, D. W. , Matthes, U. , & Kelly, P. E. (2000). Cliff ecology: Pattern and process in cliff ecosystems. Cambridge: University Press.

[ece36773-bib-0028] Lauber, K. , Wagner, G. , & Gygax, A. (2018). Flora Helvetica, illustrierte Flora der Schweiz. Bern: Haupt.

[ece36773-bib-0029] Lee, J. A. (1999). The calcicole‐calcifuge problem revisited In Callow J. A. (Ed.), Advances in Botanical Research, 29 (pp. 1–30). San Diego: Academic Press.

[ece36773-bib-0030] Lorite, J. , Serrano, F. , Lorenzo, A. , Canadas, E. M. , Ballesteros, M. , & Peñas, J. (2017). Rock climbing alters plant species composition, cover, and richness in Mediterranean limestone cliffs. PLoS One, 12, e0182414 10.1371/journal.pone.0182414 28767727 PMC5540606

[ece36773-bib-0031] March‐Salas, M. , Moreno‐Moya, M. , Palomar, G. , Tejero‐Ibarra, P. , Haeuser, E. , & Pertierra, L. R. (2018). An innovative vegetation survey design in Mediterranean cliffs shows evidence of higher tolerance of specialized rock plants to rock climbing activity. Applied Vegetation Science, 21, 289–297. 10.1111/avsc.12355

[ece36773-bib-0032] Mazenauer, D. , Holderegger, R. , Krüsi, B. , & Hepenstrick, D. (2014). Populationsentwicklung und Gefährdung von *Asplenium septentrionale* auf Findlingen im Schweizer Mittelland und Jura. Bauhinia, 25, 37–50.

[ece36773-bib-0033] Müller, S. , Rusterholz, H.‐P. , & Baur, B. (2004). Rock climbing alters the vegetation of limestone cliffs in the northern Swiss Jura Mountains. Canadian Journal of Botany, 82, 862–870. 10.1139/b04-058

[ece36773-bib-0034] Nelder, J. A. , & Wedderburn, R. W. (1972). Generalized linear models. Journal of the Royal Statistical Society. Series A (General), 135, 370–384. 10.2307/2344614

[ece36773-bib-0035] Niegl, G. (2009). Bouldering: One of the last sports defying technology? Interview with Kilian Fischhuber. Sports Technology, 2, 63–65. 10.1002/jst.116

[ece36773-bib-0036] Nuzzo, V. A. (1996). Structure of cliff vegetation on exposed cliffs and the effect of rock climbing. Canadian Journal of Botany, 74, 607–617. 10.1139/b96-077

[ece36773-bib-0037] OECD (2006). OECD Guidelines for the testing of chemicals. Paris: Organisation for Economic Cooperation and Development.

[ece36773-bib-0038] Peng, Y. , Zhang, M. , Xu, Z. Y. , Yang, T. T. , Su, Y. L. , Zhou, T. , … Lin, Y. (2020). Estimation of leaf nutrition status in degraded vegetation based on field survey and hyperspectral data. Scientific Reports, 10, 1–12. 10.1038/s41598-020-61294-7 32152356 PMC7062699

[ece36773-bib-0039] R_Core_Team , (2017). R: A language and environment for statistical computing. Vienna: R Foundation for Statistical Computing.

[ece36773-bib-0040] Ropp, R. C. (2013). Encyclopedia of the alkaline earth compounds. Oxford: Elsevier.

[ece36773-bib-0041] Rusterholz, H. P. , Müller, S. W. , & Baur, B. (2004). Effects of rock climbing on plant communities on exposed limestone cliffs in the Swiss Jura Mountains. Applied Vegetation Science, 7, 35–40. 10.1111/j.1654-109X.2004.tb00593.x

[ece36773-bib-0042] Rütti, R. , Marquer, D. , & Thompson, A. B. (2008). Tertiary tectono‐metamorphic evolution of the European margin during Alpine collison: Example of the Leventina Nappe (Central Alps, Switzerland). Swiss Journal of Geosciences, 101, 157–171. 10.1007/s00015-008-1278-9

[ece36773-bib-0043] Schmera, D. , Rusterholz, H. P. , Baur, A. , & Baur, B. (2018). Intensity‐dependent impact of sport climbing on vascular plants and land snails on limestone cliffs. Biological Conservation, 224, 63–70. 10.1016/j.biocon.2018.05.012

[ece36773-bib-0044] Shand, M. A. (2006). The chemistry and technology of magnesia. Hoboken: Wiley.

[ece36773-bib-0045] Spitale, D. , & Nascimbene, J. (2012). Spatial structure, rock type, and local environmental conditions drive moss and lichen distribution on calcareous boulders. Ecological Research, 27, 633–638. 10.1007/s11284-012-0935-7

[ece36773-bib-0046] Strumia, S. , Buonanno, M. , Aronne, G. , Santo, A. , & Santangelo, A. (2020). Monitoring of plant species and communities on coastal cliffs: Is the use of unmanned aerial vehicles suitable? Diversity, 12, 149 10.3390/d12040149

[ece36773-bib-0047] Suo, J. , Chen, S. , Zhao, Q. , Shi, L. , & Dai, S. (2015). Fern spore germination in response to environmental factors. Frontiers in Biology, 10, 358–376. 10.1007/s11515-015-1342-6

[ece36773-bib-0048] Tessler, M. , & Clark, T. A. (2016). The impact of bouldering on rock‐associated vegetation. Biological Conservation, 204, 426–433. 10.1016/j.biocon.2016.10.004

[ece36773-bib-0049] Thiel, H. , & Spribille, T. (2007). Lichens and bryophytes on shaded sandstone outcrops used for rock climbing in the vicinity of Göttingen (southern Lower Saxony, Germany). Herzogia, 20, 159–177.

[ece36773-bib-0050] Venables, W. N. , & Ripley, B. D. (2002). Modern applied statistics with S. Berlin: Springer.

[ece36773-bib-0051] Wang, W. , & Keturi, P. H. (1990). Comparative seed germination tests using ten plant species for toxicity assessment of a metal engraving effluent sample. Water, Air, and Soil Pollution, 52, 369–376. 10.1007/BF00229444

[ece36773-bib-0052] Weinbruch, S. , Dirsch, T. , Ebert, M. , Hofmann, H. , & Kandler, K. (2008). Dust exposure in indoor climbing halls. Journal of Environmental Monitoring, 10, 648–654. 10.1039/b719344k 18449402

[ece36773-bib-0053] Werdermann, P. (1993). Böhmischer Sandstein: Die schönsten Klettereien in der Tschechei. Köngen: Panico‐Alpinverlag.

[ece36773-bib-0054] Wiklund, K. , & Rydin, H. (2004). Ecophysiological constraints on spore establishment in bryophytes. Functional Ecology, 18, 907–913. 10.1111/j.0269-8463.2004.00906.x

[ece36773-bib-0055] Zhang, H. , Zhang, B. , Wei, Z. Q. , Wang, C. Z. , & Huang, Q. (2020). Lightweight integrated solution for a UAV‐borne hyperspectral imaging system. Remote Sensing, 12, 1–17. 10.3390/rs12040657

