# Supplementary material for: The distribution of climbing chalk on climbed boulders and its impact on rock‐dwelling fern and moss species
Source: Ecol Evol. 2020 Oct 1;10(20):11362–71. doi: 10.1002/ece3.6773 (PMC7593172; doi:10.1002/ece3.6773)
Supplement: Supplementary file 1 — FigS1 [file ECE3-10-11362-s001.pdf]

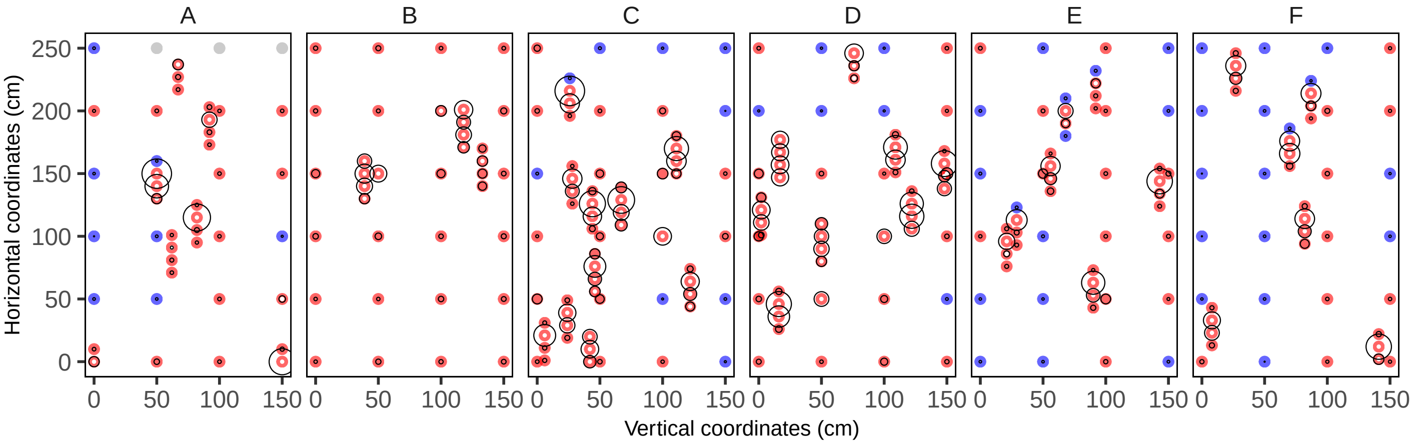

F I G U R E S1 Visualized amount of climbing chalk (magnesium) measured on six climbed boulders (ID as in Table 1). Red: elevated values above threshold level; blue: values below threshold level; gray: no data; white dots: visible climbing chalk traces at sampling points; area within black circles: proportional amount of climbing chalk measured at sampling points.
