# Supplementary material for: The distribution of climbing chalk on climbed boulders and its impact on rock‐dwelling fern and moss species
Source: Ecol Evol. 2020 Oct 1;10(20):11362–71. doi: 10.1002/ece3.6773 (PMC7593172; doi:10.1002/ece3.6773)
Supplement: Supplementary file 3 — FigS3 [file ECE3-10-11362-s003.pdf]

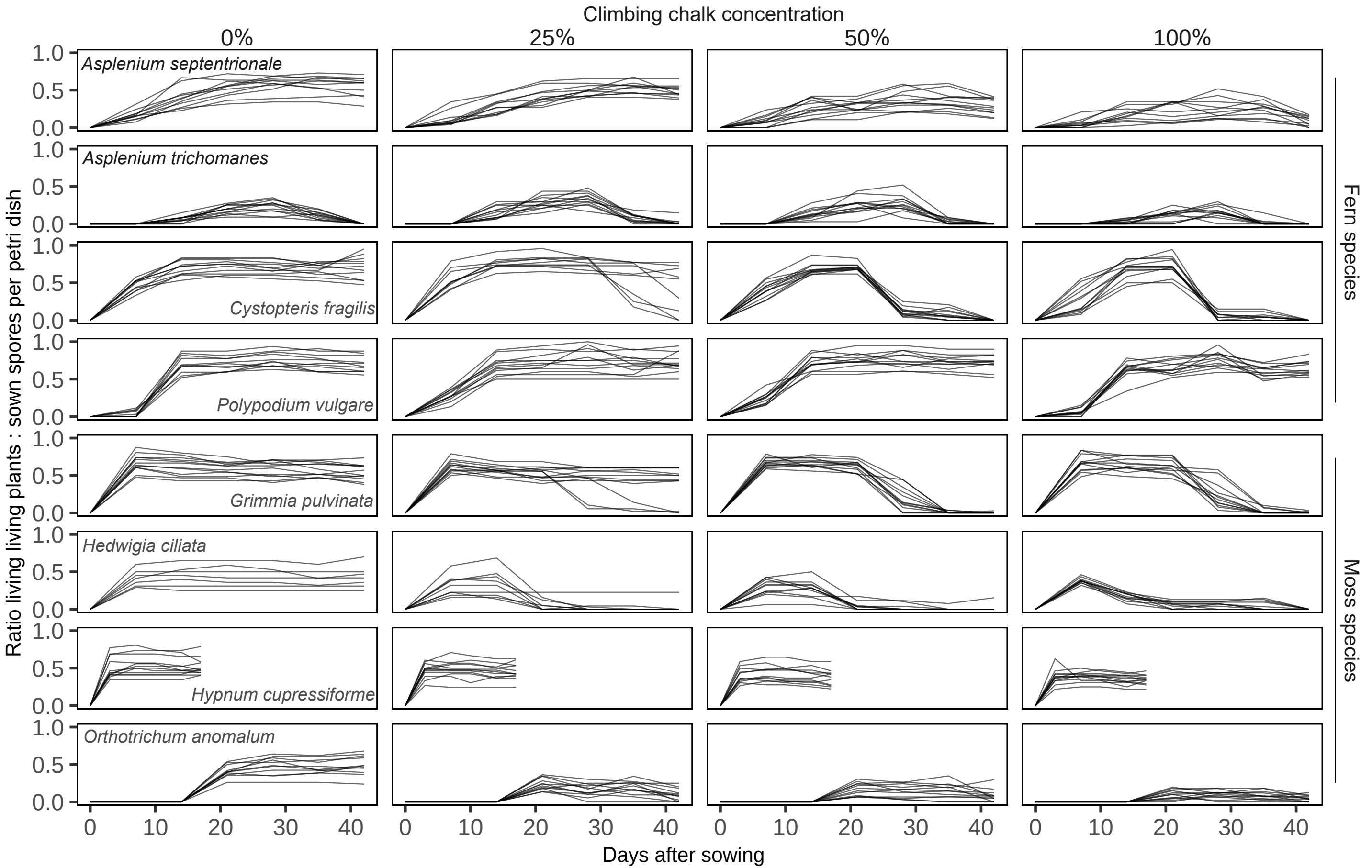

FIGURE S3 Development of four fern and four moss species on agar with four climbing chalk concentrations. Each line represents one petri dish.
